# Elevated N6-Methyladenosine RNA Levels in Peripheral Blood Immune Cells: A Novel Predictive Biomarker and Therapeutic Target for Colorectal Cancer

**DOI:** 10.3389/fimmu.2021.760747

**Published:** 2021-09-30

**Authors:** Jinye Xie, Zhijian Huang, Ping Jiang, Runan Wu, Hongbo Jiang, Chuanghua Luo, Honghai Hong, Haofan Yin

**Affiliations:** ^1^ Department of Clinical Laboratory, Zhongshan City People's Hospital, The Affiliated Zhongshan Hospital of Sun Yat-Sen University, Zhongshan, China; ^2^ Digestive Medicine Center, The Seventh Affiliated Hospital of Sun Yat-sen University, Shenzhen, China; ^3^ Department of Clinical Medical Laboratory, Guangzhou First People Hospital, School of Medicine, South China University of Technology, Guangzhou, China; ^4^ Department of Clinical Laboratory, The Seventh Affiliated Hospital of Sun Yat-sen University, Shenzhen, China; ^5^ State Key Laboratory of Oncology in South China, Collaborative Innovation Center for Cancer Medicine, Sun Yat-sen University Cancer Center, Guangzhou, China; ^6^ Department of Clinical Laboratory, The Third Affiliated Hospital of Guangzhou Medical University, Guangzhou, China

**Keywords:** N6-methyladenosine, colorectal cancer, biomarker, therapeutic target, peripheral blood

## Abstract

Effective biomarkers for the diagnosis of colorectal cancer (CRC) are essential for improving prognosis. Imbalance in regulation of N6-methyladenosine (m^6^A) RNA has been associated with a variety of cancers. However, whether the m^6^A RNA levels of peripheral blood can serve as a diagnostic biomarker for CRC is still unclear. In this research, we found that the m^6^A RNA levels of peripheral blood immune cells were apparently elevated in the CRC group compared with those in the normal controls (NCs) group. Furthermore, the m^6^A levels arose as CRC progressed and metastasized, while these levels decreased after treatment. The area under the curve (AUC) of the m^6^A levels was 0.946, which was significantly higher than the AUCs for carcinoembryonic antigen (CEA; 0.817), carbohydrate antigen 125 (CA125; 0.732), and carbohydrate antigen 19-9 (CA19-9; 0.771). Moreover, the combination of CEA, CA125, and CA19-9 with m^6^A levels improved the AUC to 0.977. Bioinformatics and qRT-PCR analysis further confirmed that the expression of m^6^A modifying regulator IGF2BP2 was markedly elevated in peripheral blood of CRC patients. Gene set variation analysis (GSVA) implied that monocyte was the most abundant m^6^A-modified immune cell type in CRC patients’ peripheral blood. Additionally, m^6^A modifications were negatively related to the immune response of monocytes. In conclusion, our results revealed that m^6^A RNA of peripheral blood immune cells was a prospective non-invasive diagnostic biomarker for CRC patients and might provide a valuable therapeutic target.

## Introduction

Colorectal cancer (CRC) is a common malignancy and the fourth leading cause of cancer-related deaths globally (1). If diagnosed in the early stage, the 5-year survival rate of CRC patients is as high as 70%–90% (2). Nevertheless, CRC patients with tumor metastases present a worse prognosis, with a 5-year survival rate of only approximately 20% (3). Furthermore, due to changes in people’s dietary and lifestyle habits, a growing number of patients with CRC are diagnosed at an advanced stage, which leads to challenging therapeutic resection of primary tumors and metastases (4).

Consequently, improving the prognosis of CRC patients largely depends on early and accurate diagnosis. At present, colonoscopy and tissue biopsy are the most efficient methods for CRC screening (5). Nonetheless, colonoscopy is an invasive procedure that can be traumatic for subjects, and the whole operation is occasionally hard to complete due to poor compliance of patients with CRC (2). Additionally, considering the invasiveness and cost of these operations, it is impractical to perform comprehensive screening as part of a general physical examination. Therefore, there is an urgent demand for more noninvasive and efficacious biomarkers for clinical diagnosis. Over recent years, the identification of blood biomarkers has become an important issue because of the pain-free operation of blood biomarkers testing (6). Blood biomarkers such as carbohydrate antigen 19-9 (CA19-9), carbohydrate antigen 125 (CA125), and carcinoembryonic antigen (CEA) are broadly applied for CRC detection (7, 8). Yet, these three biomarkers, alone or in combination, are not sufficient for diagnosing CRC due to their poor specificity and sensitivity (8, 9). Hence, there is an urgent need to optimize the diagnosis of CRC by other efficient blood biomarkers.

N6-methyladenosine (m^6^A) modification, which was encoded by the methyltransferase complex consisting of “writers”, “erasers”, and “readers”, has emerged as a critical regulator in a multitude of diseases (10, 11). The modification of m^6^A is enriched close to the 3′ untranslated terminal region (UTR) and the stop codon, thus influencing RNA transcription, processing, and translation (12, 13). Over recent years, activation of m^6^A modification has been reported in CRC tumor cells (10, 13). Upregulated m^6^A modification contributes to tumor progression by maintaining SOX2 expression in CRC cells through IGF2 mRNA binding proteins 2 (IGF2BP2)-dependent mechanisms (14, 15). Moreover, activating the glycolytic pathway by m^6^A methylation promotes CRC tumorigenesis, indicating that m^6^A modification of CRC tumor cells might become a therapeutic target (16, 17). Besides, the m^6^A-modified status of peripheral blood has been recently reported as a new promising hallmark in diabetes and gastric cancer (18, 19). Nevertheless, whether the m^6^A modification of peripheral blood RNA may act as a new diagnostic biomarker or therapeutic target for CRC remains unclear.

In this study, we examined the levels of m^6^A in peripheral blood RNA of CRC patients and NCs to assess its value as a diagnostic biomarker. We also used bioinformatics, which revealed that elevated m^6^A levels were mainly associated with monocytes and suppressed their immune response, indicating that m^6^A modifications of peripheral blood immune cells might become a therapeutic target for CRC.

## Materials and Methods

### Human Samples

The Institutional Review Board of Zhongshan People’s Hospital approved this retrospective study (IRB number: K2020-20) on March 20, 2020. Between March 2020 and June 2021, peripheral blood samples from 105 CRC patients and 64 NCs who had no history of basic or chronic diseases were collected from the Zhongshan People’s Hospital, using EDTA anticoagulation tubes. Whole blood (0.5 ml) and 1 ml of red blood cell lysate (TIANGEN, Beijing, China) were mixed and centrifuged. The precipitate was taken and dissolved with 1 ml TRIzol to stabilize RNA, after which the mixed samples were stored at −80°C for no longer than 6 months. All CRC patients were diagnosed on the basis of the histopathology by biopsy or endoscopic examination, and informed consent was obtained for all participants. A total of 105 CRC patients’ peripheral blood samples were collected at the time of diagnosis before surgery or radiochemotherapy. Of these, peripheral blood was collected for the first time on admission and for the second time 14 days after surgery in 33 CRC patients. Ethics approval was obtained from the Ethics Committee of the Zhongshan People’s Hospital. The clinical and biological characteristics of the patients are described in **Table 1**.

**Table 1:** Correlation between the levels of m^6^A and clinicopathological characteristics in CRC.

Characteristics	No. of patients	Peripheral blood m^6^A levels % (mean ± SD)	*p-*value
Age
≤60	57	0.268 ± 0.057	0.649
>60	48	0.273 ± 0.040
Gender
Female	36	0.276 ± 0.064	0.386
Male	69	0.267 ± 0.043
Clinical stage
I	6	0.243 ± 0.031	0.682
II	20	0.263 ± 0.031
III	31	0.260 ± 0.048
IV	26	0.302 ± 0.063
T classification
T1–T2	15	0.268 ± 0.040	0.739
T3–T4	64	0.274 ± 0.056
N classification
N0	29	0.273 ± 0.066	0.933
N1–N2	50	0.272 ± 0.046
N classification
N0–N1	57	0.269 ± 0.056	0.291
N2	22	0.283 ± 0.047
M classification
M0	57	0.260 ± 0.041	<0.001
M1	26	0.302 ± 0.063
Differentiation
Poor	14	0.273 ± 0.030	0.975
Moderate/Well	70	0.273 ± 0.056
Tumor budding
Bd1–Bd2	12	0.262 ± 0.043	0.861
Bd3	16	0.259 ± 0.042
HER2 expression
Negative	26	0.256 ± 0.040	0.368
Positive	26	0.267 ± 0.044
KRAS genotyping
Wild type	10	0.277 ± 0.042	0.360
Mutation type	7	0.299 ± 0.053
BRAF genotyping
Wild type	17	0.279 ± 0.049	0.600
Mutation type	3	0.295 ± 0.031
CEA (ng/ml)
<5	44	0.265 ± 0.040	0.202
≥5	54	0.278 ± 0.057
CA125 (ng/ml)	
<35	68	0.269 ± 0.043	0.298
≥35	30	0.280 ± 0.063
CA19-9 (ng/ml)
<35	66	0.271 ± 0.054	0.742
≥35	32	0.275 ± 0.041

### RNA Isolation and qRT-PCR

Total RNA was extracted using TRIzol (Thermo Scientific, MA, USA) according to the manufacturer’s protocol. First-strand cDNA synthesis was performed using 500 ng of total RNA, and the qRT-PCR analysis system was performed using iQ SYBR Green Supermix (Accurate Biology, Changsha, China) and the iCycler Real-time PCR Detection System (Bio-Rad, California, USA). β-actin was used for normalization. Primers of targeted genes are listed in **Supplementary Table S1**.

### Monocyte Isolation

Peripheral blood mononuclear cells (PBMCs) were isolated from peripheral blood samples from CRC patients and normal subjects *via* density gradient centrifugation. Whole blood was collected in EDTA tubes. The blood was diluted 1:1 with PBS free of calcium and magnesium. PBMCs were obtained by Ficoll density gradient isolation (Stemcell Technologies, Cologne, Germany). From the freshly isolated PBMCs, CD14^+^ monocytes were isolated using the EasySep Human Monocyte Isolation Kit (Stemcell Technologies, Cologne, Germany).

### RNA m^6^A Quantification

The m^6^A levels in total RNA were measured using EpiQuik m^6^A RNA Methylation Quantification Kit (Colorimetric) (Epigentek, New York, USA) according to the manufacturer’s protocol. RNA (200 ng) was added to assay wells covered with binding solution. Capture antibody solution, detection antibody solution, and enhancer solution were sequentially added to assay wells with diluted concentration, as specified in the manufacturer’s instructions. Developer solution and stop solution were added to the color reaction, after which the absorbance of each well at a wavelength of 450 nm was measured. The m^6^A levels were calculated based on the standard curve.

### Bioinformatics Analysis

The RNA-seq data and clinical data of the peripheral blood of CRC and NCs were obtained from GEO (Gene Expression Omnibus) databases (GSE164191). Differential expression analysis was conducted by “limma” package of R studio (3.6.1) software. Gene set variation analysis (GSVA) was performed to estimate m6A modified pathways based on GO molecular function N6 methyladenosine containing RNA binding gene set and **Figure 4B** listed genes. Immune infiltrates of peripheral blood were estimated *via* MCP-counter method. Gene Set Enrichment Analysis (GSEA) was manipulated to predict the GO biological process gene sets of the Molecular Signature Database v7.4 (http://www.broadinstitute.org/gsea/msigdb) based on IGF2BP1/IGF2BP2/IGF2BP3 high and low expressed phenotype. A leading edge analysis was performed by GSEA 4.1.0 to elucidate key genes related to selected genes sets. EnrichmentMap plugin in Cytoscape 3.8.2 software was utilized with the following parameters: *p*-value cutoff = 0.05; similarity coefficient cutoff = 0.5. The protein–protein interaction (PPI) networks were constructed using The Search Tool for the Retrieval of Interacting Genes (STRING), which is a publicly available software for assessing the interaction between proteins and proteins (https://string-db.org/).

### Statistical Analysis

The variability of the data, which was presented as the SD (mean ± SD), was assessed with unpaired Student’s *t* test between two groups for normally distributed data. Otherwise, the data were analyzed by nonparametric Mann–Whitney test. Paired *t*-tests were used to analyze the effects of treatment on m^6^A levels. For multiple groups, significant differences were determined using one-way ANOVA. Pearson correlation analysis was conducted to determine the correlation between GSVA scores and immune infiltrates. Forest plot of multivariate logistic regression analysis was performed to access risk indicators associated with CRC diagnosis. Statistical significance was defined at *p* < 0.05.

## Results

### The m^6^A RNA Levels of Peripheral Blood Immune Cells in CRC Patients and NCs

**Figure 1:**
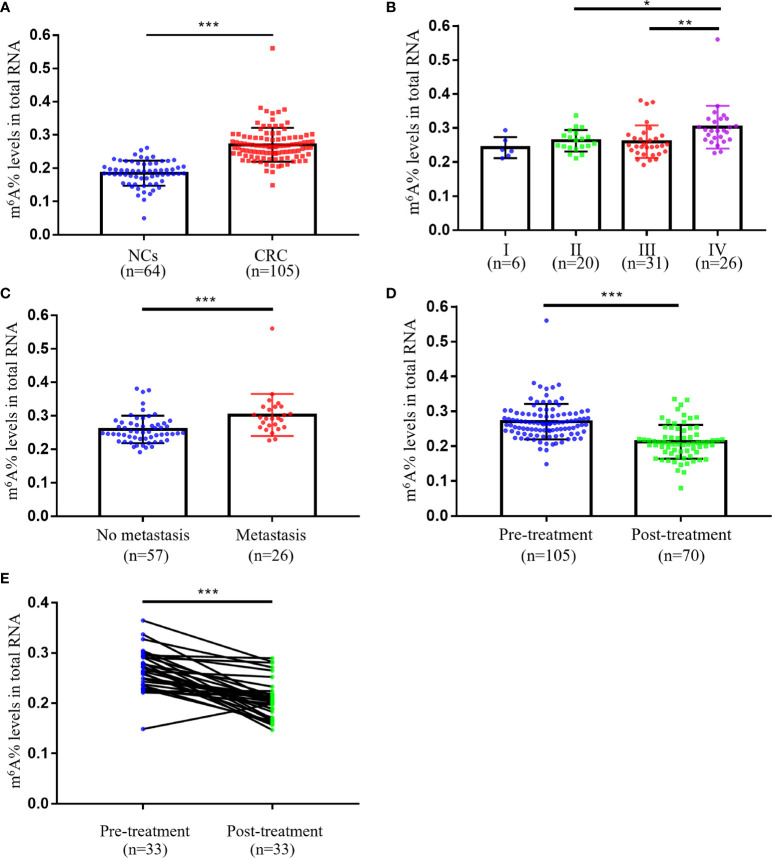
The m^6^A RNA levels of peripheral blood immune cells in CRC patients and NCs. **(A)** The m^6^A levels of peripheral blood RNA in NCs (*n* = 64) and CRC patients (*n* = 105). **(B)** The m^6^A levels of peripheral blood RNA at different clinical stages of CRC patients (stage I, *n* = 6; stage II, *n* = 20; stage III, *n* = 31; stage IV, *n* = 26). **(C)** Comparison of m^6^A levels of peripheral blood RNA between CRC patients with (*n* = 26) and without (*n* = 57) metastasis. **(D)** Comparison of m^6^A levels of peripheral blood RNA between CRC patients with (*n* = 70) and without (*n* = 105) treatment. **(E)** The m^6^A levels of peripheral blood RNA in CRC patients (*n* = 33) before and after 14 days of treatment. Bars represent the mean ± SD of the results from replicate measurements; *p < 0.05, **p < 0.01 and ***p < 0.001.

First, we analyzed the m6A levels of total RNA in NCs (*n* = 64) and CRC patients (*n* = 105) so as to evaluate the status of m6A modification in peripheral blood immune cells. The m^6^A levels in peripheral blood immune cells were remarkably increased in patients with CRC (0.271 ± 0.051) than in NCs (0.185 ± 0.038; **Figure 1A**). Furthermore, statistical analyses of the relationship between the m^6^A levels and clinicopathological features of CRC are performed in **Table 1**. Our data indicated that the m^6^A levels correlated with M classification (*p* < 0.001), but not with clinical stage, T classification, N classification, differentiation, tumor budding, as well as other common CRC tumor markers, including CEA, CA125, and CA19-9 (**Table 1**). As shown in **Figure 1B**, the levels of m^6^A were dramatically elevated in the stage IV group (*n* = 26, 0.302 ± 0.063) than in stage I (*n* = 6, 0.243 ± 0.031), II (*n* = 20, 0.263 ± 0.031), or III groups (*n* = 31, 0.260 ± 0.048). In addition, CRC patients with distant tumor metastasis (*n* = 26, 0.302 ± 0.063) had apparently increased m^6^A levels compared to those without distant metastasis (*n* = 57, 0.259 ± 0.041; **Figure 1C**). These results suggested that peripheral blood m^6^A RNA levels could partially distinguish the various pathological stages in patients with CRC.

To elucidate whether m^6^A could be used to assess treatment status in CRC patients, we compared the m^6^A levels of peripheral blood between the pre-treatment group and post-treatment group. The obtained results demonstrated that m^6^A levels were markedly reduced in the post-treatment group (**Figure 1D**). We also observed significant changes in m^6^A levels before and after surgery (14 days) in 33 CRC patients, indicating that m^6^A RNA levels of peripheral blood immune cells could be used as a promising indicator for post-treatment follow-up (**Figure 1E**).

### Clinical Utility for CEA, CA125, CA19-9, and the m^6^A RNA Levels of Peripheral Blood Immune Cells to Diagnose CRC Patients

**Figure 2:**
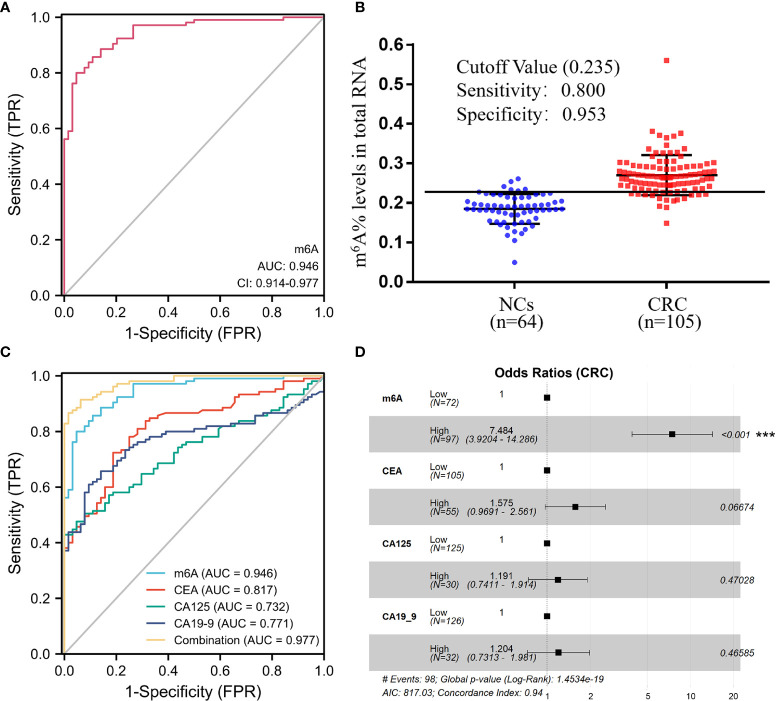
Clinical utility for CEA, CA125, CA19-9, and the m^6^A RNA levels of peripheral blood immune cells to diagnose CRC patients. **(A, B)** ROC curve **(A)** and cutoff value **(B)** of the m^6^A levels of peripheral blood RNA in NCs (*n* = 64) and CRC patients (*n* = 105). **(C)** ROC curve of the m^6^A levels of peripheral blood RNA compared and combined diagnosis with CEA, CA125, and CA19-9. **(D)** Forest plot of multivariate logistic regression analysis demonstrated that the m6A levels were an independent factor associated with CRC diagnosis; ***p < 0.001.

We plotted ROC curves to further assess the diagnostic capability of m^6^A RNA levels of peripheral blood immune cells for CRC. The area under the curve (AUC) of m^6^A was up to 0.946 (95% CI, 0.914–0.977), indicating that m^6^A levels could differentiate CRC patients from NCs (**Figure 2A**). Also, the optimum m^6^A cutoff value was 0.235 (specificity, 0.953; sensitivity, 0.800; **Figure 2B**). Impressively, the diagnostic ability of m^6^A was superior to the usual CRC blood biomarkers, such as CEA, CA125, and CA19-9, with AUCs of 0.817, 0.732, and 0.771, respectively (**Figure 2C** and **Table 2**). Moreover, the ROC curve for the multivariate combination of m^6^A, CEA, CA125, and CA19-9 increased the AUC to 0.977 (95% CI, 0.961–0.994; **Figure 2C**). Furthermore, the forest plot of multivariate logistic regression analysis demonstrated that the m^6^A levels were an independent factor associated with CRC diagnosis (**Figure 2D**). Taken together, these results clarified that the m^6^A RNA levels of peripheral blood immune cells presented satisfactory diagnostic utility for CRC patients.

**Table 2:** Sensitivity and specificity of the diagnostic value of various markers alone and in combination.

Marker	Sensitivity	Specificity	AUC	95% CI
m^6^A	0.800	0.953	0.946	0.914–0.977
CEA	0.724	0.812	0.817	0.754–0.881
CA125	0.476	0.953	0.732	0.659–0.806
CA19-9	0.657	0.859	0.771	0.700–0.842
m^6^A+CEA+CA125+CA19-9	0.914	0.938	0.977	0.961–0.994

### Expressions and Diagnostic Values of IGF2BP1, IGF2BP2, and IGF2BP3 in Peripheral Blood Immune Cells of CRC Patients

**Figure 3:**
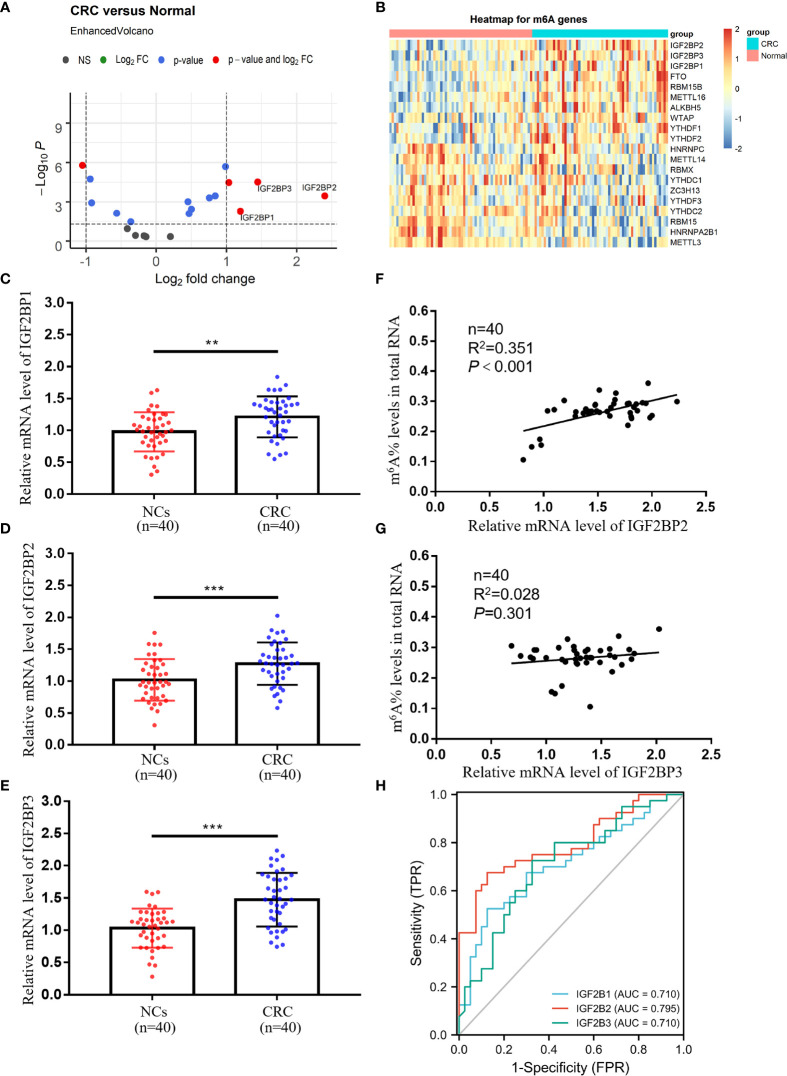
Expressions and diagnostic values of IGF2BP1, IGF2BP2, and IGF2BP3 in peripheral blood immune cells of CRC patients. **(A)** Screening key molecules related to m^6^A modification in peripheral blood of CRC patients (*n* = 59) compared to normal subjects (*n* = 62) by limma differential analysis. **(B)** Heatmap of key molecules related to m6A modification in peripheral blood of CRC patients. **(C–E)** qRT-PCR analysis of IGF2BP1 **(C)**, IGF2BP2 **(D)**, and IGF2BP3 **(E)** mRNA expression levels in peripheral blood of NCs and CRC patients. **(F, G)** Correlation between the levels of IGF2BP2/IGF2BP3 and m^6^A in peripheral blood of CRC patients. **(H)** ROC curves of the IGF2BP1, IGF2BP2, and IGF2BP3 mRNA expression levels in peripheral blood of CRC patients. Bars represent the mean ± SD of the results from replicate measurements; **p < 0.01, ***p < 0.001.

To screen for core molecules that regulate m^6^A modifications in peripheral blood immune cells RNA, we analyzed the GSE164191 dataset, containing RNA-seq data on peripheral blood leukocytes of CRC patients and normal subjects. Surprisingly, members of the IGF2BP family (IGF2BP1, IGF2BP2, and IGF2BP3) were the most dramatically altered molecules in the methyltransferase complex consisting of “writers”, “erasers”, and “readers” (**Figures 3A, B**). Meanwhile, the strongest increase in IGF2BP2 was observed in CRC patients, suggesting a potentially vital role in m^6^A modification of peripheral blood immune cells (**Figures 3A, B**). qRT-PCR analysis also proved significantly higher expression of IGF2BP1, IGF2BP2, and IGF2BP3 in CRC patients compared to normal subjects (**Figures 3C–E**). We further discovered a relationship between the levels of m^6^A and the expressions of IGF2BP2, but no correlation with the expressions of IGF2BP1 and IGF2BP3 (**Figures 3F, G** and **Supplementary Figure 1**). The AUCs of IGF2BP1, IGF2BP2, and IGF2BP3 were 0.710, 0.795, and 0.710, respectively (**Figure 3H**). Their AUCs were similar to common CRC blood biomarkers CEA, CA125, and CA19-9 but still smaller than the AUC of m^6^A. Collectively, IGF2BP2 in peripheral blood immune cells was a potentially valuable diagnostic biomarker for CRC associated with m^6^A modification.

### Correlation Between Immune Infiltrating Cell Types and m^6^A Modification in Peripheral Blood Immune Cells of CRC Patients

**Figure 4:**
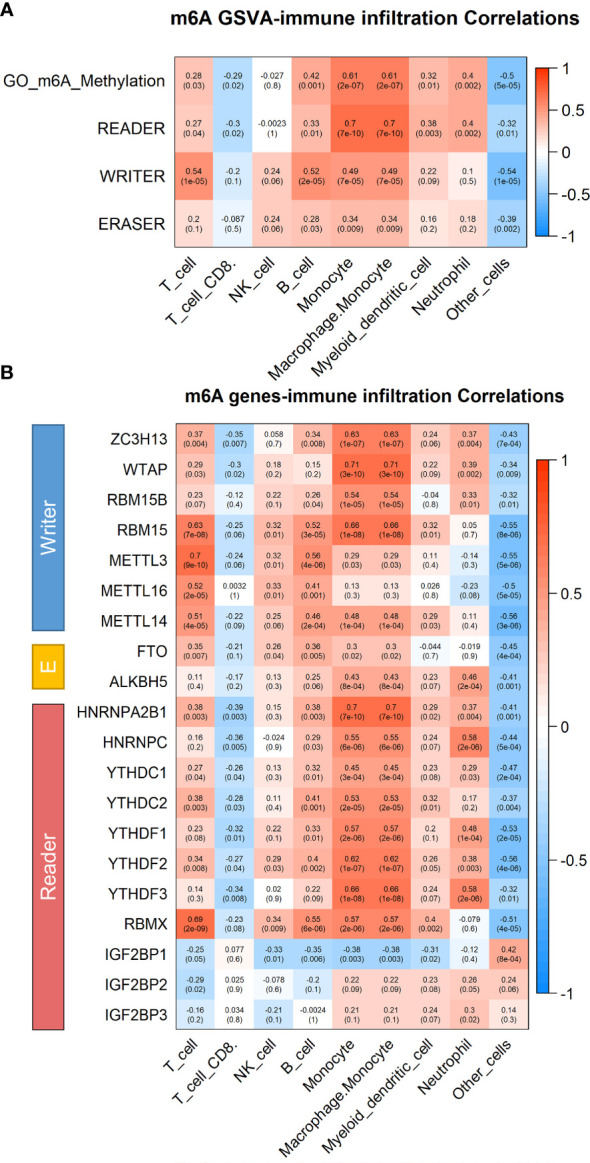
Correlation between immune infiltrating cell types and m^6^A modification in peripheral blood immune cells of CRC patients. **(A)** Heatmap of correlation between immune infiltrating cell types and m^6^A modification pathways in peripheral blood of CRC patients by Gene set variation analysis by GSVA (*n* = 59). **(B)** Heatmap of correlation between immune infiltrating cell types and m^6^A modification related gene in peripheral blood of CRC patients by GSVA.

To further elucidate the specific immune cells associated with elevated m^6^A levels of peripheral blood in CRC patients, we analyzed the GSE164191 database by GSVA. The obtained results suggested that the methyltransferase complexes, consisting of “writer”, “eraser”, and “reader”, all exhibited the strongest positive correlation with monocytes infiltrating (**Figure 4A**). Detection of monocytes isolated from peripheral blood of CRC patients and normal subjects also revealed that monocytes from CRC patients possessed higher levels of m^6^A (**Supplementary Figure 2**). Meanwhile, infiltration of monocytes was also markedly correlated with IGF2BP2 expression, consistent with the results in **Figure 3** regarding the importance of IGF2BP2 in m^6^A modifications (**Figure 4B**). In conclusion, monocytes resulted as the specific immune cells most strongly associated with upregulated m^6^A levels of peripheral blood immune cells in CRC patients.

### IGF2BP2 Involved in the Immune Response of Monocytes in Peripheral Blood of CRC Patients

**Figure 5:**
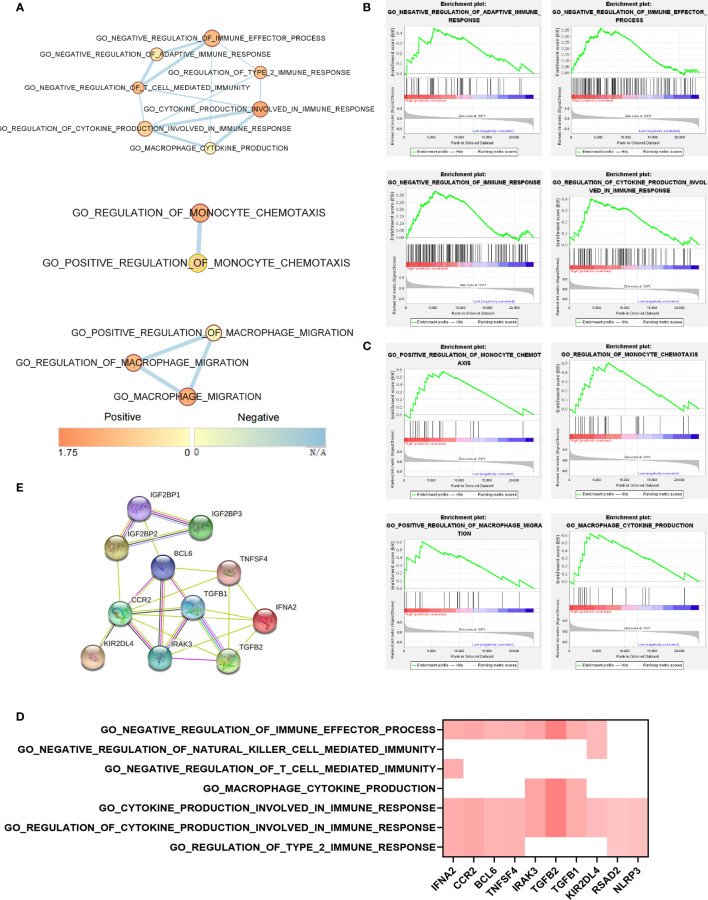
IGF2BP2 involved in the immune response of monocytes in peripheral blood of CRC patients. **(A)** EnrichmentMap pathways network revealed overlaps among IGF2BP2 high-expressed phenotype enriched pathways relating to immunity in peripheral blood of CRC patients. Nodes are colored by Enrichment Score, and edges are sized on the basis of the number of genes shared by the connected pathways. **(B)** GSEA indicated that IGF2BP2 was negatively correlated with the immune response of monocytes. **(C)** GSEA indicated that IGF2BP2 was positively correlated with monocyte chemotaxis and cytokine production. **(D)** Leading edge analysis of their intersection genes indicates the vital genes shared by the IGF2BP2 high-expressed phenotype associated with the immune response of monocytes. **(E)** STRING database analysis revealed that IGF2BP2 interacted with the above vital genes related to the immune response of monocytes.

The function of IGF2BP2 in the monocytes of the peripheral blood of CRC patients was investigated using the EnrichmentMap plugin in Cytoscape 3.8.2 software. The corresponding association network showed that the IGF2BP2 high-expression phenotype presented a robust positive association between several monocyte immune response pathways (**Figure 5A**). GSEA was applied to predict the biological processes of monocytes in peripheral blood based on IGF2BP2 expression. Likewise, high IGF2BP2 expression was mainly enriched in the immune response pathways, such as “Negative regulation of immune effector process”, “Regulation of monocyte chemotaxis”, and “Cytokine production” (**Figures 5B, C**). Additionally, the results of leading edge analysis identified the intersection of important genes associated with the immune response pathways (**Figure 5D**). Meanwhile, the PPI networks structured by the STRING database suggested that IGF2BP2 may interact with the above vital genes (**Figure 5E**). IGF2BP1 and IGF2BP3 also performed approximately the same immune functions as IGF2BP2 in monocytes (**Supplementary Figure 3**). Taken together, IGF2BP2 exerted an essential role in the immune response of peripheral blood monocytes of CRC patients.

## Discussion

Most patients are already at an advanced stage by the time they are diagnosed with CRC, which substantially contributes to the poor prognosis (4). Hence, improving the prognosis of CRC patients depends on an early and accurate diagnosis. However, the currently used clinical tumor biomarkers for CRC such as CEA, CA125, and CA19-9 are not specific or sensitive enough to detect CRC patients (7, 9). Therefore, optimizing the diagnosis of CRC with other validated biomarkers is of urgent importance. The present study identified the m^6^A status of peripheral blood immune cells as a novel marker for CRC screening. In addition, it might also serve as a new target for CRC treatment.

Despite a growing body of reports that have linked m^6^A dysregulation to various cancers, the role of m^6^A modifications in CRC tumor tissues remained controversial (10, 20). Stimulating m^6^A modification promotes β-catenin translation to drive the epithelial–mesenchymal transition of CRC cells, while some studies found that m6A regulation suppresses proliferation and metastasis (15, 21, 22). Our research revealed for the first time that the m^6^A RNA levels of peripheral blood immune cells were dramatically higher in patients with CRC than in healthy subjects (**Figure 1A**). Our results demonstrated that m^6^A RNA was more strongly modified in peripheral blood immune cells of CRC, yet m^6^A modification in CRC tumor tissue needs to be further explored. Additionally, the m^6^A status of peripheral blood immune cells was substantially elevated in CRC patients with distant metastases compared to those without metastases, implying that it could also discriminate if the tumor had metastasized (**Figures 1B, C**). Although the m^6^A levels were reduced in treated CRC patients, more clinical samples were requested to determine whether they could be used as an indicator of oncologic efficacy, such as relapse and drug resistance (**Figures 1D, E**). It has been discussed that the m^6^A levels might be applied as a biomarker for gastric cancer, but the regulation of m^6^A modification in different tumors varied significantly (18, 23). Therefore, it is worthwhile to investigate further whether the m^6^A levels had diagnostic value in other tumors.

CEA, CA125, and CA19-9 are widely used in physical screening for CRC (9). Nevertheless, due to their poor specificity and sensitivity, these three biomarkers alone or in combination are not sufficient to diagnose CRC (7). As shown in **Figure 2**, the AUC for m^6^A to differentiate CRC patients from healthy subjects was 0.946 (95% CI, 0.914–0.977), which was significantly higher than the AUC for CEA (0.817; 95% CI, 0.754–0.881), CA125 (0.732; 95% CI, 0.659–0.806), and CA19-9 (0.771; 95% CI, 0.700–0.842). The combination of CEA, CA125, and CA19-9 with m^6^A further increased the AUC to 0.977 (95% CI, 0.961–0.994). Besides, forest plots from multiple logistic regression analysis showed that the m^6^A levels were an independent risk factor associated with the diagnosis of CRC compared to these common tumor biomarkers (**Figure 2D**). Our study presented a considerable challenge to the value of these tumor biomarkers.

“Writers”, “erasers”, and “readers” together formed the methyltransferase complex responsible for m^6^A modification. Wilms tumor 1-associated protein (WTAP), Methyltransferase-like 3 (METTL3), and METTL14 were classified as “writers” catalyzing the formation of m^6^A (24–26). AlkB homolog 5 (ALKBH5) and Fat mass and obesity-associated protein (FTO) represented “erasers”, meaning they could induce selective removal of methylation code from the target mRNA (27, 28). “Readers” were able to decode m^6^A modification, comprising YT521-B homology domain-containing protein (YTHDF) as well as IGF2BP families (16, 29). m^6^A modifications altered the expression of target genes and changed the consequent biological features (30). To further understand the role of the elevated m^6^A levels in CRC tumor progression, we screened for the most variable “writers”, “erasers”, and “readers” in CRC peripheral blood immune cells by limma differential analysis. Members of the IGF2BP family (IGF2BP1, IGF2BP2, and IGF2BP3), which belonged to “readers”, were the most markedly changed molecules in the methyltransferase complex (**Figure 3**). Simultaneously, IGF2BP2 revealed the greatest increase, thus suggesting a potentially crucial role in peripheral blood immune cell m^6^A modification (**Figure 3**). Unlike other readers, IGF2BPs acted as a unique family of m^6^A readers that target a multitude of mRNA transcripts and enhance the conservation and stability of their candidate mRNAs in an m^6^A-dependent way (14, 15, 31). Our study further demonstrated that elevated IGF2BP2 might interact with several essential genes to negatively regulate immunity, such as cytokine production and chemotaxis (**Figure 5** and **Supplementary Figure 3**). Although we found that increased IGF2BPs expression combined with elevated m^6^A levels affected cancer immunity in CRC, we have not yet clarified the mechanism of increased IGF2BPs, which is also the biggest limitation of the current study. Taken together, m^6^A modification and IGF2BPs expression were likely to be novel targets for CRC treatment, but further *in vivo* experimental studies are required.

Previous studies reported that elevated m^6^A levels of peripheral blood in patients with gastric cancer might be due to downregulation of FTO and ALKBH5, which belonged to “erasers” (18). Our qRT-PCR results also revealed a slight downregulation of FTO and ALKBH5 in peripheral blood cells of CRC patients, partially explaining the increased m^6^A levels (**Supplementary Figure 4**). Other unknown methylases and demethylases may also be involved in the changes of m^6^A levels that deserved further exploration (32). Additionally, monocytes were identified as the immune cells most strongly associated with the increased regulation of upregulated m^6^A levels in peripheral blood of CRC patients (**Figure 4**). It has been noted that the presence of a large number of m^6^A-modified infiltrating immune cells in the tumor tissue microenvironment promotes tumor progression (33, 34). Furthermore, imbalanced m^6^A regulation strongly conferred immune disruption and tumor evasion, primarily by affecting immune cell migration, rather than apoptosis or survival (35). These observations were generally consistent with our findings in peripheral blood immune cells. Moreover, the number of monocytes in the CD14^+^CD16^+^HLA-DR^hi^ subpopulation of patient’s peripheral blood was found to be the most accurate predictor of progression-free survival and overall survival after receiving PD-1 inhibitor therapy (36). Whether the subset of monocytes with elevated m^6^A levels had a similar role in tumor immunotherapy to the CD14^+^CD16^+^HLA-DR^hi^ subset deserves further investigation.

In conclusion, the highlights of our research were the first identification of m^6^A RNA levels in peripheral blood immune cells as a novel biomarker for the diagnosis of CRC and the provision of a new strategy for the treatment of CRC by targeting m^6^A levels or IGF2BPs expression in peripheral blood immune cells.

## Data Availability Statement

The original contributions presented in the study are included in the article/**Supplementary Material**. Further inquiries can be directed to the corresponding authors.

## Ethics Statement

The studies involving human participants were reviewed and approved by the Ethics Committee of the Zhongshan People’s Hospital. The patients/participants provided their written informed consent to participate in this study.

## Author Contributions

HY, HH, and CL conceived and designed this study. JX, ZH, and PJ performed the experiments and analyzed the data. RW and HJ contributed to the data analysis and discussion. All authors contributed to the article and approved the submitted version.

## Funding

This study was supported by the fund from the National Nature Science Foundation of China (81900775; 81902693); Educational Commission of Guangdong Province (2017KTSCX155); Guangdong Basic and Applied Basic Research Foundation (2019A1515011318); Natural Science Foundation of Guangdong Province (2018A030310298); the Science Foundation of Guangzhou First People’s Hospital (Q2019004; KYQD0046); China Postdoctoral Science Foundation (2019M662991); Key Medical and Health Projects of Zhongshan City (2020K0012); Guangzhou Science and Technology Planning Project (202102020142).

## Conflict of Interest

The authors declare that the research was conducted in the absence of any commercial or financial relationships that could be construed as a potential conflict of interest.

## Publisher’s Note

All claims expressed in this article are solely those of the authors and do not necessarily represent those of their affiliated organizations, or those of the publisher, the editors and the reviewers. Any product that may be evaluated in this article, or claim that may be made by its manufacturer, is not guaranteed or endorsed by the publisher.

## Supplementary Material

Supplementary Figure 1 Correlation between the levels of IGF2BP1 and m^6^A in peripheral blood of CRC patients. Absence of correlation between the m^6^A levels and IGF2BP1 expression.

Supplementary Figure 2 The m^6^A levels of monocytes isolated from peripheral blood of CRC patients and normal subjects. The m^6^A levels of monocytes isolated from CRC patients was higher than those in monocytes from normal subjects.

Supplementary Figure 3 IGF2BP1 and IGF2BP3 expression are negatively associated with several immune response pathways. **(A, B)** EnrichmentMap pathways network exhibited connectivity among IGF2BP1 **(A)** and IGF2BP3 **(B)** high-expressed phenotype enriched pathways relating to immunity response in peripheral blood of CRC patients. **(C, D)** GSEA indicated that IGF2BP1 **(C)** and IGF2BP3 **(D)** were negatively correlated with the immune response of monocytes.

Supplementary Figure 4 Expressions of FTO and ALKBH5 in peripheral blood RNA of CRC patients. **(A, B)** Q-PCR analysis of FTO **(A)** and ALKBH5 **(B)** mRNA expression levels in peripheral blood of NCs and CRC patients.
