# Supplementary figures and images for: Elevated N6-Methyladenosine RNA Levels in Peripheral Blood Immune Cells: A Novel Predictive Biomarker and Therapeutic Target for Colorectal Cancer
Source: Front Immunol. 2021 Sep 30;12:760747. doi: 10.3389/fimmu.2021.760747 (PMC8515146; doi:10.3389/fimmu.2021.760747)

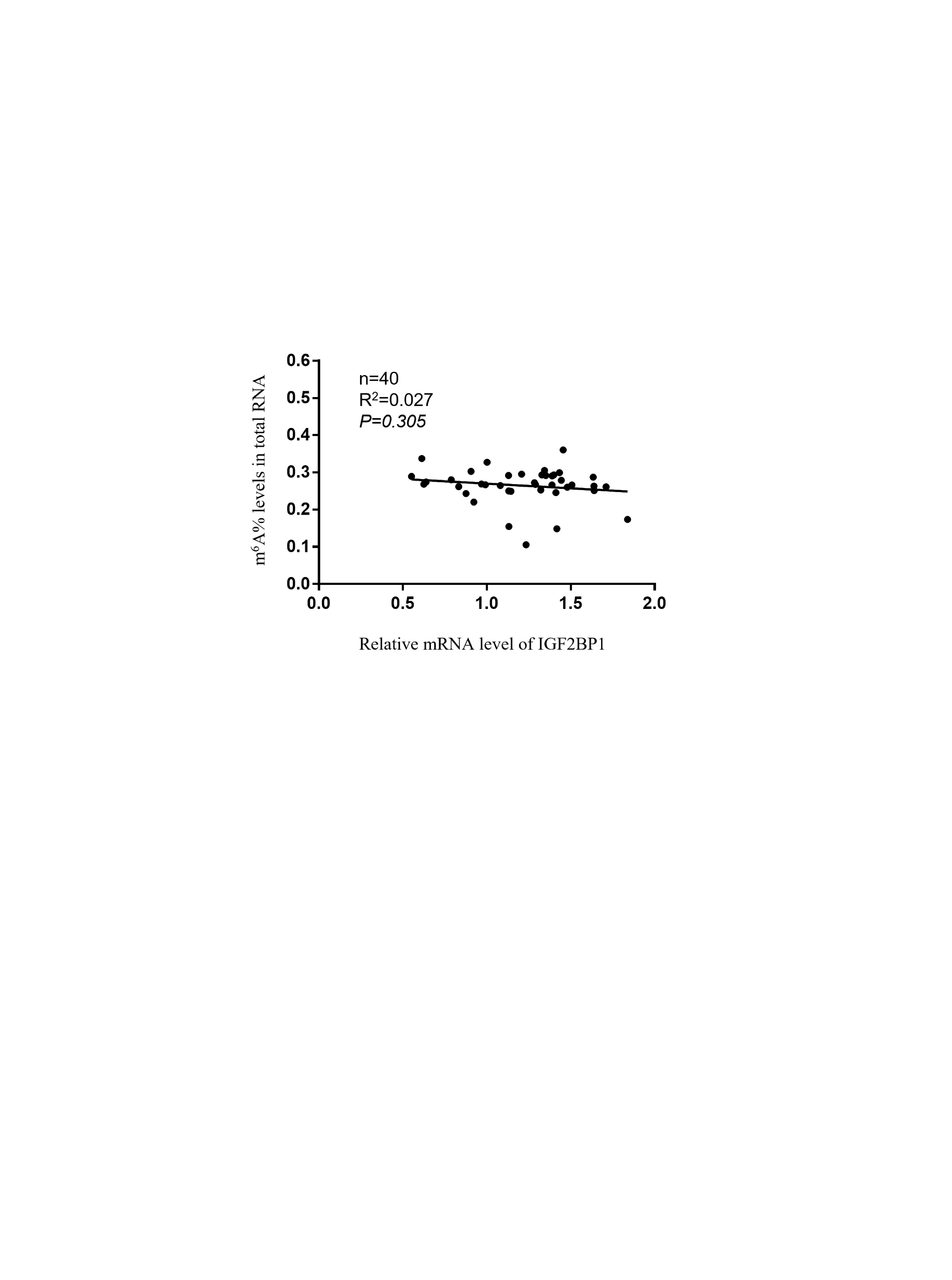

Supplement: Supplementary Figure 1 — Correlation between the levels of IGF2BP1 and m6A in peripheral blood of CRC patients. Absence of correlation between the m6A levels and IGF2BP1 expression. [file Image_1.tif]

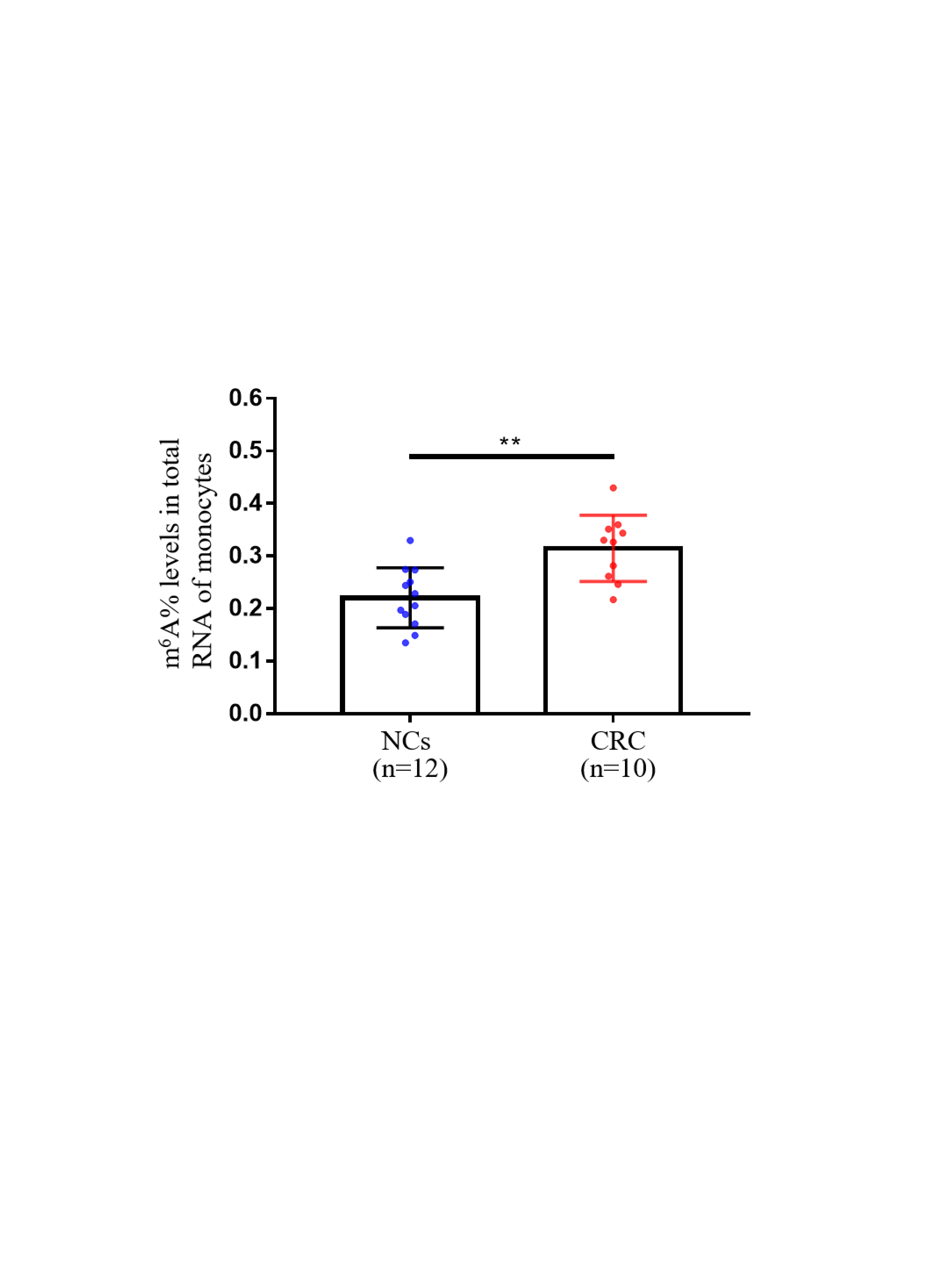

Supplement: Supplementary Figure 2 — The m6A levels of monocytes isolated from peripheral blood of CRC patients and normal subjects. The m6A levels of monocytes isolated from CRC patients was higher than those in monocytes from normal subjects. [file Image_2.tif]

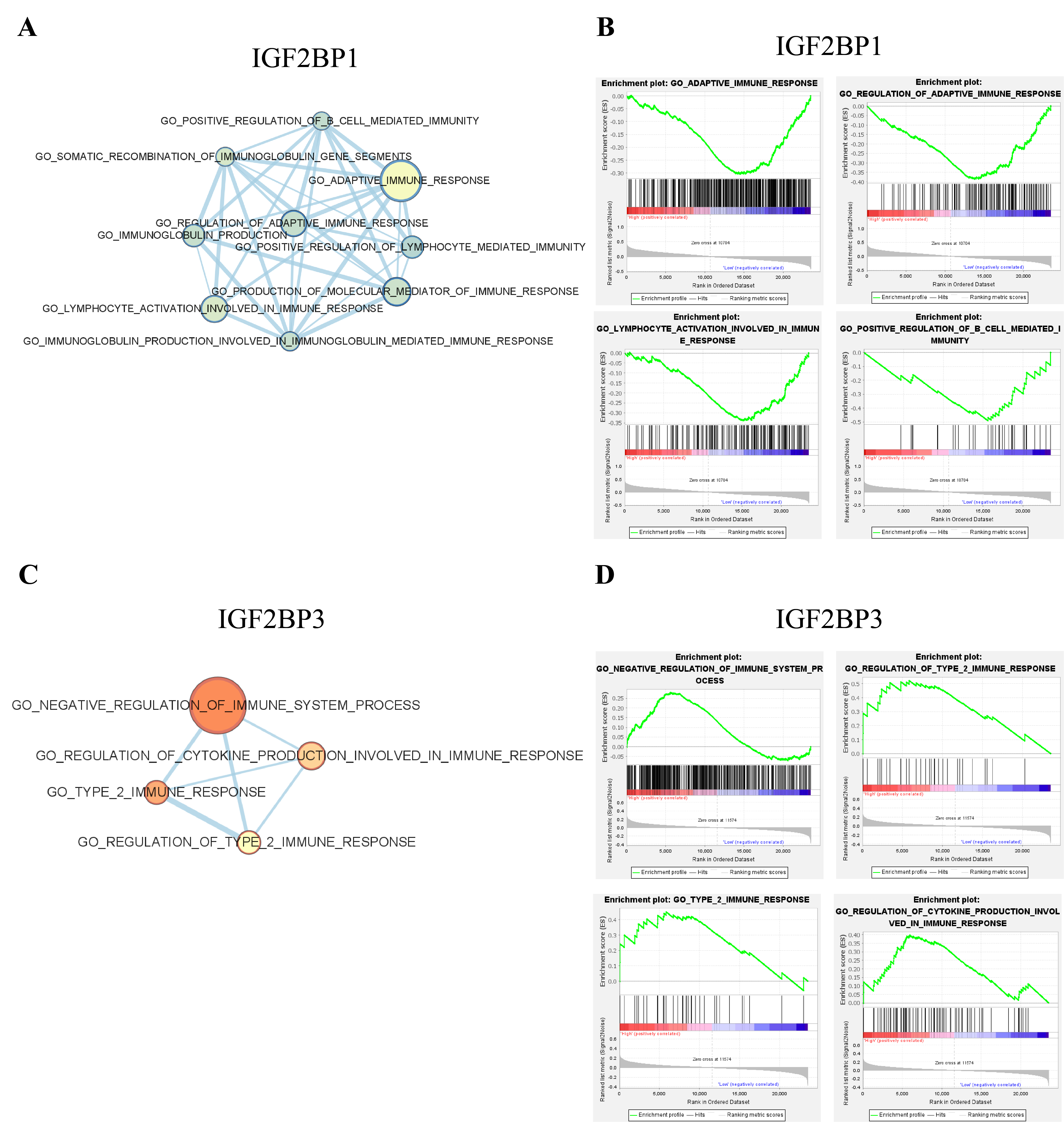

Supplement: Supplementary Figure 3 — IGF2BP1 and IGF2BP3 expression are negatively associated with several immune response pathways. (A, B) EnrichmentMap pathways network exhibited connectivity among IGF2BP1 (A) and IGF2BP3 (B) high-expressed phenotype enriched pathways relating to immunity response in peripheral blood of CRC patients. (C, D) GSEA indicated that IGF2BP1 (C) and IGF2BP3 (D) were negatively correlated with the immune response of monocytes. [file Image_3.tif]

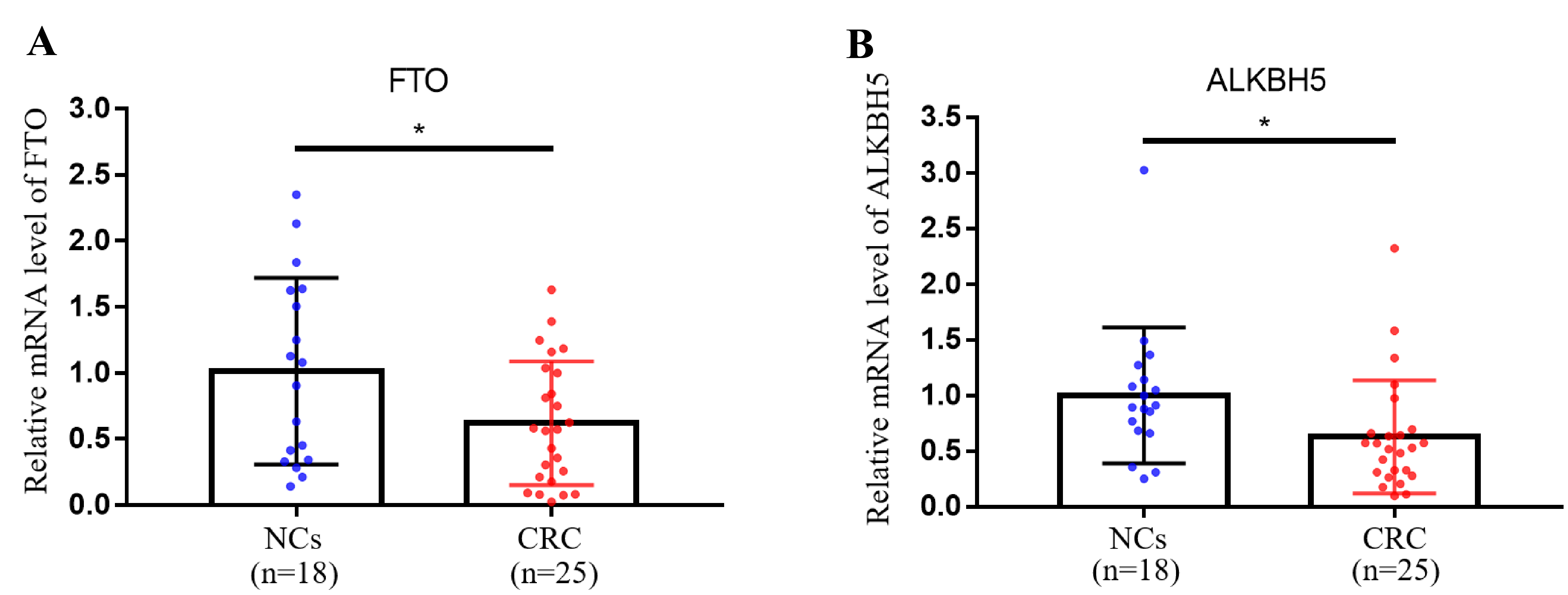

Supplement: Supplementary Figure 4 — Expressions of FTO and ALKBH5 in peripheral blood RNA of CRC patients. (A, B) Q-PCR analysis of FTO (A) and ALKBH5 (B) mRNA expression levels in peripheral blood of NCs and CRC patients. [file Image_4.tif]
