# Supplementary material for: Elevated N6-Methyladenosine RNA Levels in Peripheral Blood Immune Cells: A Novel Predictive Biomarker and Therapeutic Target for Colorectal Cancer
Source: Front Immunol. 2021 Sep 30;12:760747. doi: 10.3389/fimmu.2021.760747 (PMC8515146; doi:10.3389/fimmu.2021.760747)
Supplement: Supplementary file 5 [file Table_1.docx]

**Supplementary** **Table. 1 Primer sequence**

| Primer | Sequence (5’ to 3’) |
| --- | --- |
| METTL3-F | CTATCTCCTGGCACTCGCAAGA |
| METTL3-R | GCTTGAACCGTGCAACCACATC |
| METTL14-F | AGTGCCGACAGCATTGGTG |
| METTL14-R | GGAGCAGAGGTATCATAGGAAGC |
| ZC3H13-F | AAAGGAGGTTTCACCAGAAGTG |
| ZC3H13-R | CGCTTCGGAGATTTGCTAGAC |
| ALKBH5-F | CCAGCTATGCTTCAGATCGCCT |
| ALKBH5-R | GGTTCTCTTCCTTGTCCATCTCC |
| FTO-F | TTGGACGGTACAGATATGGAACATTTT |
| FTO-R | TCTTTTAGTTTCTTTGCCTTTGGGGAT |
| IGF2BP1-F | GCGGCCAGTTCTTGGTCAA |
| IGF2BP1-R | TTGGGCACCGAATGTTCAATC |
| IGF2BP2-F | AGTGGAATTGCATGGGAAAATCA |
| IGF2BP2-R | CAACGGCGGTTTCTGTGTC |
| IGF2BP3-F | TATATCGGAAACCTCAGCGAGA |
| IGF2BP3-R | GGACCGAGTGCTCAACTTCT |
| β-actin-F | ACTCTTCCAGCCTTCCTTC |
| β-actin-R | ATCTCCTTCTGCATCCTGTC |
